# Blood pressure pattern among blood donors exposed to SARS‐CoV‐2 in Luanda, Angola: A retrospective study

**DOI:** 10.1002/hsr2.1498

**Published:** 2023-08-16

**Authors:** Cruz S. Sebastião, Euclides Sacomboio, Ngiambudulu M. Francisco, Edson K. Cassinela, António Mateus, Zinga David, Victor Pimentel, Joana Paixão, Jocelyne Neto de Vasconcelos, Joana Morais

**Affiliations:** ^1^ Instituto Nacional de Investigação em Saúde (INIS) Luanda Angola; ^2^ Centro de Investigação em Saúde de Angola (CISA) Caxito Angola; ^3^ Instituto de Ciências da Saúde (ICISA) Luanda Angola; ^4^ Global Health and Tropical Medicine (GHTM) Instituto de Higiene e Medicina Tropical (IHMT) Lisbon Portugal; ^5^ Centro de Estudos Investigação Cientifica e Pós‐graduação (CEIP) Luanda Angola; ^6^ Centro Nacional de Investigação Científica (CNIC) Luanda Angola

**Keywords:** ABO/Rh blood groups, Angola, hypertension, Luanda, SARS‐CoV‐2

## Abstract

**Background and Aims:**

SARS‐CoV‐2 infection is a public health concern. Several aspects related to the pattern of infection remain unclear. This study aimed to investigate the blood pressure pattern among blood donors exposed to SARS‐CoV‐2 in Luanda, Angola, a sub‐Saharan African country.

**Methods:**

We performed a retrospective analysis containing 343 blood donors from December 2019 to September 2020. Parametric tests compared means while *χ* ^2^ and logistic regression checked features associated with high blood pressure and were considered significant when *p* < 0.05.

**Results:**

The mean age of blood donors was 32.2 ± 8.81 years (ranging from 18 to 61 years) and 93% of the men's gender. Overall, 4.7% of the studied population had been exposed to SARS‐CoV‐2. High blood pressure prevalence increased from unexposed to exposed SARS‐CoV‐2 (6.7%–18.8%, *p* = 0.071). SARS‐CoV‐2 exposure increase systole (131 ± 12.2 mmHg to 136 ± 14.2 mmHg, *p* = 0.098), diastole (79.9 ± 9.53 mmHg to 84.2 ± 12.7 mmHg, *p* = 0.086), pulse in beats per minute (72.0 ± 11.1 to 73.7 ± 8.50, *p* = 0.553), and decrease donating time (6.31 ± 3.72 min to 5.48 ± 1.61 min, *p* = 0.371). Chances of having high blood pressure were high [OR: 3.20 (95% confidence interval [CI]: 0.85–12.1), *p* = 0.086] in exposed SARS‐CoV‐2. Donors exposed to SARS‐CoV‐2 with abnormal donation time increased from the donor up to 40 years to over 40 years (from 35.7% to 50%, *p* = 0.696). The mean systolic, diastolic, and pulse pressure were higher for non‐O donors (*p* > 0.05). A significant link was observed, between the Rhesus factor and blood pressure status (*p* = 0.032).

**Conclusion:**

We showed important variations in blood pressure indices of the Angolan population exposed to SARS‐CoV‐2. Older age and non‐O blood groups appear to be important biological factors for SARS‐CoV‐2 infection, as well as the risk of developing cardiovascular disease after or during SARS‐CoV‐2 exposure. Further studies assessing the impact on cardiovascular functions with ongoing or long‐term SARS‐CoV‐2 exposure in individuals from resource‐limited countries should be considered.

## 1 INTRODUCTION

SARS‐CoV‐2 is currently considered a major concern for global public health due to its large social and economic repercussions as well as impacts on the stability of health systems, mainly in low‐ and middle‐income countries (LMICs).^1^, ^2^ Currently, more than 600 million individuals were infected and more than 6.5 million deaths have been reported globally, of these, more than 100 thousand infections and about 2000 deaths were reported in Angola.^3^ As expected, LMICs tend to be less responsive to the COVID‐19 pandemic, especially in the aspects of diagnosis, dissemination, and molecular evolution at the community level.^4^, ^5^, ^6^, ^7^ On the other hand, high‐income countries have shown high rates of infection, spread, mortality, and molecular variability of the SARS‐CoV‐2, mainly due to the installation of the virus in the older population, as reported by several studies, which, in turn, these aged infected cannot, from an immunological point of view, fight the infection naturally.^8^ As a result, high mortality has been observed in the adult population or with a history of any chronic disease, such as hypertension, diabetes, or even cardiovascular disease that is becoming increasingly prevalent in sub‐Saharan Africa (SSA) although limited data still exist from Africa on the effects of these noncommunicable diseases (NCDs) on COVID‐19.^9^, ^10^, ^11^, ^12^ In recent decades, resource‐limited countries have witnessed a significant shift towards increased blood pressure, and yet only one in three people are aware of their hypertension status ensuring their blood pressure is controlled resulting in increased costs to the local healthcare system.^13^ A previous study noted discordant patterns in the relationship between COVID‐19 and cardiovascular disease or hypertension in sub‐Saharan African countries.^14^ In the context of a global pandemic such as COVID‐19, with the increase in cases and deaths reported worldwide, many people living with NCDs in African countries might find it more difficult to access care as most of the available resources have been diverted to focus on the pandemic which has caused disruptions in NCD management, with significant implications for NCDs and the continuity of care in Sub‐Saharan Africa after the era of COVID‐19.^15^

The medical and scientific community have continually made strenuous efforts to gain a deeper understanding of the disease, mainly by identifying the pattern of SARS‐CoV‐2 infection, transmission, and COVID‐19 severity in populations from different settings.^8^ Even though, as the virus spreads in different regions and populations with different features, several aspects related to the pattern of infection remain unclear and need to be studied, such as the distribution pattern of blood pressure indices in a population exposed to SARS‐CoV‐2 short or long term.^16^ Previous studies have shown that high systolic blood pressure on hospital admission is an important risk factor in models that predict the outcome of patients with COVID‐19.^17^, ^18^, ^19^, ^20^

To the best of our knowledge, there is no published study assessing the blood pressure pattern and possibly biological and nonbiological factors that may be related to changes in blood pressure in the exposed SARS‐CoV‐2 population from Angola. Therefore, this study aimed to investigate for the first time the putative profile of blood pressure in an asymptomatic population exposed to SARS‐CoV‐2 and residents in Luanda, the capital city of Angola, a sub‐Saharan African country, to present strategies to expand clinical and epidemiological management as well as to reduce the risk of developing cardiovascular disease in individuals exposed to SARS‐CoV‐2 in Angola.

## 2 METHODS

### 2.1 Study design and setting

This was a retrospective study that included information from clinical records of 343 blood donors assessed as healthy for blood donation at the Instituto Nacional de Sangue and Clínica Girassol, both health units located in Luanda, the capital city of Angola, between December 2019 and September 2020. The main inclusion criterion used for the selection of study participants was to be considered able to donate blood after a previous clinical evaluation, which implied the seronegativity of infectious diseases such as HIV, HBV, HCV, and syphilis. The survey was conducted at Instituto Nacional de Investigação em Saúde (INIS). The INIS is a public institution of the Angolan Ministry of Health (MoH), which is developing research in numerous areas of health as well as its determining factors, intending to contribute to the strengthening of public health policy in Angola. Being a retrospective study, the informed agreement was waived by the National Ethics Committee of the Angolan MoH.

### 2.2 Data acquisition and description

A structured questionnaire was used to gather data such as age, gender, and donation duration in minutes from databases of the healthcare units. Frozen blood plasma samples were used to test the infectious disease markers such as anti‐SARS‐CoV‐2 antibody screening. Specimens were thawed and an estimated 5 mL aliquot of the blood plasma was used for the qualitative detection of IgM/IgG antibodies against SARS‐CoV‐2 with enzyme‐linked fluorescence assays (bioMérieux SA), commercially available, following the manufacturer's instructions. Positive and negative control supplied by the manufacturer has been incorporated into all the reactions. Findings were grouped as follows: past infection (IgG+/IgM–) and recent infection (IgG–/IgM+ or IgG+/IgM+). ABO/Rh blood‐group phenotypes determination was conducted using blood grouping antisera and diagnostic kits (Lorne Laboratories Limited), according to the manufacturer's instructions.^21^ The following ABO blood groups A, B, and AB were categorized as non‐O. We used the Trucare KD‐558BR arm manometer (Andon Health CO., Ltd) for the measurement of systolic, diastolic, and pulse blood pressure.^22^ The entire blood pressure monitor process, as well as the interpretation of systolic, diastolic, and pulse findings, were conducted following the manufacturer's instructions.^22^ For this study, we considered the CDC guidelines and findings from previous studies, which show that the blood donation candidate can only be approved when at least the maximum systolic blood pressure is below 140 mmHg and the diastolic below 90 mmHg.^23^, ^24^ Consequently, high systolic blood pressure was taken into consideration when the values were higher than 140 mmHg, while diastolic pressure was considered elevated when the values were higher than 90 mmHg. Candidates who have had high blood pressure above 140/90 mmHg have been grouped into the high or abnormal blood pressure category. Normal blood donation time in this study was considered to be between 6 and 10 min while the pulse pressure was between 60 and 100 beats per minute.

### 2.3 Statistical analysis

Statistical analyses were performed in SPSS v28 (IBM SPSS Statistics). The descriptive analysis was introduced as frequencies and percentages. The normal data distribution was presented as mean and standard deviation (SD). Independent‐sample *T* tests were used for comparing mean values. The categorical variables were dichotomized and analyzed with the *χ* ^2^ test and univariate logistic regression with an appropriate 95% confidence interval (CI). The reported *p* value is two‐tailed and was considered to be statistically significant when *p* < 0.05.

## 3 RESULTS

### 3.1 Blood pressure patterns among exposed and nonexposed to SARS‐CoV‐2

The pattern of blood pressure in blood donors exposed to and unexposed to SARS‐CoV‐2 is shown in Table 1. A total of 343 blood donors fulfilled the inclusion criteria and formed part of the analyses. The mean age of blood donors was 32.2 ± 8.81 years, which varies from 18 to 61 years, being the men's gender prevailing at 93% (319/343). Concerning the ABO/Rh blood groups, blood group O represented approximately 63.6% (218/343) and the Rh+ factor was in the majority with 97.4% (334/343) in this studied population (results not shown). Overall, 4.7% (16/343) of blood donors enrolled in the study had a history of being exposed to SARS‐CoV‐2, either past (4.7%) or recent (3.5%) infections. The mean in systolic pressure (131 ± 12.2 mmHg to 136 ± 14.2 mmHg, *p* = 0.098), diastolic pressure (79.9 ± 9.53 mmHg to 84.2 ± 12.7 mmHg, *p* = 0.086), and pulse in beast per minutes (72.0 ± 11.1 to 73.7 ± 8.50, *p* = 0.553) increases with exposure to SARS‐CoV‐2, even though any statistical relevance (*p* > 0.05). Also, a nonsignificant reduction was noted in the duration of donating time in minutes (6.31 ± 3.72 to 5.48 ± 1.61, *p* = 0.371). No significant relationship has been observed between exposure to SARS‐CoV‐2 and blood pressure status, pulse, or duration of blood donation (*p* > 0.05). Nevertheless, the prevalence of blood donors with high blood pressure has risen from unexposed to exposed to SARS‐CoV‐2 (6.7%–18.8%, *p* = 0.071). The mean values of systolic, diastolic, and pulse blood pressure, were higher for donors with past infection, although no statistical significance (*p* > 0.05). Also, no significant relationship was found between the past infection caused by SARS‐CoV‐2 with blood pressure status, pulse, or duration of blood donation. Conversely, marked increases in mean systolic (131 ± 12.2 mmHg to 143 ± 9.96 mmHg, *p* = 0.022) and diastolic (80.0 ± 9.64 mmHg to 89.8 ± 11.6 mmHg, *p* = 0.024) blood pressure were observed in the donors with recent SARS‐CoV‐2 infection. Additionally, a statistically significant relationship was found between recent SARS‐CoV‐2 infection and blood pressure status, being checked as an increase significantly by around six times (from 6.8% to 40%, *p* = 0.005). Moreover, the prevalence of donors with abnormal donation duration increased with recent infection by around two times, from 31% to 60%, although this is not significant (*p* = 0.161). Chances of a blood donor exposed to the SARS‐CoV‐2 having high blood pressure were high [OR: 3.20 (95% CI: 0.85–12.1), *p* = 0.086] in comparison to unexposed donors. Furthermore, the probability of the donation duration being abnormal was high [OR: 1.34 (95% CI: 0.48–3.79), *p* = 0.578] in the exposure to SARS‐CoV‐2 infection.

**Table 1:** Blood pressure distribution pattern among exposed and nonexposed subjects to SARS‐CoV‐2 in Luanda, Angola.

Independent variables	*N* (%)	Anti‐SARS‐CoV‐2 positivity			Past infection			Recent infection			Univariate analysis	
		No (%)	Yes (%)	*p* Value	No (%)	Yes (%)	*p* Value	No (%)	Yes (%)	*p* Value	OR (95% CI)	*p* Value
Overall	343 (100)	327 (95.3)	16 (4.70)		331 (96.5)	16 (4.70)		331 (96.5)	12 (3.50)			
Blood pressure, mmHg												
SBP, mean ± SD	131 ± 12.3	131 ± 12.2	136 ± 14.2	0.098	131 ± 12.2	132 ± 14.4	0.807	131 ± 12.2	143 ± 9.96	**0.022**	‐	‐
DBP, mean ± SD	80.1 ± 9.72	79.9 ± 9.53	84.2 ± 12.7	0.086	80.0 ± 9.59	83.0 ± 13.1	0.295	80.0 ± 9.64	89.8 ± 11.6	**0.024**	‐	‐
Distribution												
Normal (≤140/90)	318 (92.7)	305 (93.3)	13 (81.3)	0.071	307 (92.7)	11 (91.7)	0.887	315 (93.2)	3 (60.0)	**0.005**	1.00	
Abnormal (>140/90)	25 (7.30)	22 (6.70)	3 (18.8)		24 (7.30)	1 (8.30)		23 (6.80)	2 (40.0)		3.20 (0.85 – 12.1)	0.086
Pulse pressure, BPM												
Mean ± SD	72.1 ± 11.0	72.0 ± 11.1	73.7 ± 8.50	0.553	72.1 ± 11.0	71.6 ± 10.7	0.869	72.1 ± 11.0	74.4 ± 7.86	0.637	‐	‐
Distribution												
Normal (60–100)	311 (90.7)	295 (90.2)	16 (100)	0.189	300 (90.6)	11 (91.7)	0.904	306 (90.5)	5 (100)	0.470	1.00	
Abnormal (<60 or >100)	32 (9.30)	32 (9.80)	0 (0.00)		31 (9.40)	1 (8.30)		32 (9.50)	0 (0.00)		0.00 (0.00–0.00)	0.998
Donation duration, minutes												
Mean ± SD	6.27 ± 3.65	6.31 ± 3.72	5.48 ± 1.61	0.371	6.24 ± 3.61	7.20 ± 4.61	0.371	6.30 ± 3.67	4.43 ± 1.17	0.258		
Distribution												
Normal (5–10)	236 (68.8)	226 (69.1)	10 (62.5)	0.577	228 (68.9)	8 (66.7)	0.871	234 (69.2)	2 (40.0)	0.161	1.00	
Abnormal (<5 or >10)	107 (31.2)	101 (30.9)	6 (37.5)		103 (31.1)	4 (33.3)		104 (30.8)	3 (60.0)		1.34 (0.48–3.79)	0.578

*Note*: Past infection (IgG+/IgM–) and recent infection (IgG–/IgM+ or IgG+/IgM+). The bold number was statistically significant in the χ^2^ test or independent‐sample *T* tests (*p* < 0.05).

Abbreviations: BPM, beats per minute; DBP, diastolic blood pressure; SBP, systolic blood pressure.

### 3.2 Biological factors linked to the blood pressure pattern among exposure to SARS‐CoV‐2

All SARS‐CoV‐2 exposure donors in the current study were men (100%, 16/16). Of these, 87.5% have been aged up to 40 years, while the other 12.5% were over 40‐year‐old. The mean values of systolic, diastolic, and pulse blood pressure, were higher for donors over 40 years when compared with donors up to 40 years. No donor was having an abnormal pulse. By contrast, all donors with high blood pressure were aged up to 40 years, even with that, no significant relationship was found between age distribution and abnormal blood pressure. Mean values of duration of donation time were lower in donors over 40‐year‐old when compared with the younger donors up to 40 years. No significant relationship was found between the age group and duration of donation, even though the prevalence of donors exposed to SARS‐CoV‐2 with abnormal donation time increased from the donor up to 40 years to donors over 40 years (from 35.7% to 50%, *p* = 0.696). Regarding the ABO/Rh blood group, the non‐O blood groups (56.3%, 9/16) and Rh+ (93.8%, 15/16) donors, were predominant. The mean systolic, diastolic, and pulse pressure were higher for non‐O blood group donors, even though there was no statistically significant difference between the mean values (*p* > 0.05). Moreover, no link was noted between O and non‐O blood groups with the blood pressure status, pulse, or duration of donation (*p* > 0.05). Even though the Rh+ factor is the predominant, high mean values that apply to systolic and diastolic blood pressure, as well as donation duration, were observed among Rh‐ blood donors. Moreover, a statistically significant link was observed, between the Rh factor and blood pressure status (*p* = 0.032).

## 4 DISCUSSION

To the best of our knowledge, this study provides the first possible evidence that individuals exposed to SARS‐CoV‐2 infection are prone to developing cardiovascular disease in Luanda, the capital city of Angola. Generally, elevations in blood pressure values have been identified as an independent factor in the development of cardiovascular disease or end‐stage renal disease. We showed that individuals exposed to SARS‐CoV‐2 have blood pressure levels of around 136/84 mmHg. Although it is within the accepted values as normal,^25^ these values were high in comparison to the average of the healthy population and not exposed to SARS‐CoV‐2 in Angola, which was around 131/80. These findings showed that the need for interventions to prevent cardiovascular or kidney disease should focus not only on individuals who have blood pressure above 140/90, which is globally considered high but also pay attention to individuals with normal high blood pressure values, especially those exposed to viral infectious diseases, such as the SARS‐CoV‐2 because they present high mean values when compared to the mean values of the population not exposed to viral infectious diseases. These results also emphasize that blood pressure quintile (I. Lowest, II. Second, III. Third, IV. Fourth, and V. Highest) definition studies in the Angolan population exposed or not to viral infectious diseases as well as the population with or without the presence of chronic diseases should be carried out in different regions, to assist Angolan clinicians in decision‐making to prevent the progression of cases of cardiovascular or renal diseases in the population.^25^, ^26^

It is worth mentioning that individuals with active or recent infection with positive IgM antibodies against SARS‐CoV‐2 had a mean blood pressure of around 143/90 mmHg. Furthermore, the prevalence of individuals with high blood pressure status increases significantly (6.8%–40%, *p* = 0.005) with the presence of active or recent SARS‐CoV‐2 infection (Table 1). We cannot rule out the possibility that these individuals already had a history of high blood pressure before they were exposed to SARS‐CoV‐2.^27^ Despite this, these findings showed on the one hand that individuals with a previous history of high blood pressure have a higher risk of becoming infected or developing severe COVID‐19, as reported by previous studies.^11^, ^28^, ^29^, ^30^ Indeed, a study carried out by Wang and colleagues, showed that 58% (21/66) of patients who required admission to intensive care were patients with hypertension, compared to 22% (22/51) of hypertensive patients who did not develop COVID‐19 severe (*p* < 0.001).^28^ On the other hand, our findings showed that SARS‐CoV‐2 infection could induce unfavorable cardiovascular clinical outcomes, starting with a significant rise in the values of the systolic pressure (from 131 ± 12.2 mmHg to 143 ± 9.96 mmHg, *p* = 0.022), diastolic pressure (from 80.0 ± 9.64 mmHg to 89.8 ± 11.6 mmHg, *p* = 0.024), and pulse (from 72.1 ± 11.0 mmHg to 74.4 ± 7.86 mmHg, *p* = 0.637), especially in individuals with active or recent SARS‐CoV‐2 infection. In addition to the statistically significant relationship observed between recent SARS‐CoV‐2 infection and high blood pressure (*p* = 0.005), we also observed that the risk of an exposed individual with recent or past SARS‐CoV‐2 infection to developing high blood pressure is 3.2 times (95% CI: 0.85–12.1, *p* = 0.086), compared to the population not exposed to SARS‐CoV‐2. These results suggest that the clinical staff should closely monitor blood pressure indices in an individual with a recent infection or do a clinical follow‐up of blood pressure in individuals exposed to SARS‐CoV‐2. We showed that the increase in blood pressure also implies an increase in the pulse blood pressure and as a result of the increase in the pulse blood pressure, we will have reduced blood donation time (Table 1). As we expected, we observed a significant borderline (*p* = 0.060) between blood donation time and blood pressure status, where almost half (48%, 12/35) of individuals with high blood pressure had abnormal blood donation time compared to 30% (95/318) of blood donors with normal blood pressure, who also had abnormal donation time (results not shown). Furthermore, we showed that individuals exposed to SARS‐CoV‐2 might have a blood donation time 1.34 times higher (95% CI: 0.48–3.79, *p* = 0.578), compared to the unexposed population. Identification of individuals at high risk of developing cardiovascular disease from the analysis of the time of blood donation needs to be explored in future studies and the information generated will be crucial to immediately assist in the management of blood donors with abnormal donation time. Also, the relationship between the psychosocial status of the blood donor with changes in blood pressure and the time of donation needs to be explored in the future.

Previous studies have shown that there appears to be an association between ABO/Rh blood groups and SARS‐CoV‐2 infection.^31^, ^32^, ^33^ The observation that the population exposed to SARS‐CoV‐2 with blood group O did not exceed 50% (Table 2) might be in agreement with results reported by Cheng and colleagues, where they showed that blood group O was associated with reduced susceptibility to SARS infection.^34^ Generally, studies have shown that people with blood group O could be the least likely to be infected with SARS‐CoV‐2, despite being the group that most needed treatment in the hospital and artificial respiration compared to other non‐O blood groups.^35^, ^36^, ^37^, ^38^ We previously demonstrated a lower SARS‐CoV‐2 positivity in blood group O individuals as well as a lower risk of infection compared to non‐O individuals.^39^ The affinity between SARS‐CoV‐2 with the cells of the respiratory or gut system as a function of the host's ABO/Rh blood group is not yet clarified and is the subject of further investigation in the future. Furthermore, has no scientific evidence showing whether the blood pressure of the population exposed to SARS‐CoV‐2 changes according to ABO/Rh blood groups. Even though, we already expected that blood donors over 40 years of age would have the highest mean blood pressure or pulse and the shortest donation time (Table 2), in agreement with what was described by other authors who reported an increase in blood pressure with increasing age.^23^, ^24^ However, it is worth noting that advanced age (over 40 years) has previously been described as an independent risk factor for SARS‐CoV‐2 infection and severity, even in Angola, as shown by our research team.^4^, ^39^, ^40^ In addition, this study reports that adults over 40 years of age are more likely to contract viral infectious diseases and also have a high chance of developing high blood pressure with a potential risk for an unfavorable cardiovascular or renal clinical outcome. In terms of ABO/Rh blood groups, it is worth mentioning at the first stage that different from the Rh+ factor that represented over 50% of the Angola population, the blood group O despite being the most prevalent, no longer represents more than 50% of the population (Table 2). This differs from the prevalence described in a study carried out in 1973 by Spielmann et al.^41^ where blood group O represented between 54% and 59% of the Angolan population, suggestings a decrease of blood group O in the population over the years. Indeed, a recent study carried out by our research team, showed a significant increase in non‐O blood groups, with special attention to blood group B,^39^ which, in addition to increasing its prevalence, has been associated with the risk of arterial hypertension in the Angolan population.^42^, ^43^

**Table 2:** Biological factors linked to the blood pressure pattern among exposure subjects to SARS‐CoV‐2 in Luanda, Angola.

Independent variables	Gender	Age group			ABO blood group			Rh blood type		
	Male (%)	≤40 years (%)	>40 years (%)	*p* Value	Non‐O (%)	O (%)	*p* Value	Rh– (%)	Rh+ (%)	*p* Value
Overall	16 (100)	14 (87.5)	2 (12.5)		9 (56.3)	7 (43.8)		1 (6.30)	15 (93.8)	
Blood pressure, mmHg										
SBP, mean ± SD	136 ± 14.2	135 ± 14.6	139 ± 14.9	0.775	137 ± 12.3	134 ± 17.2	0.714	150 ± 0.00	135 ± 14.1	0.313
DBP, mean ± SD	84.2 ± 12.7	83.7 ± 13.5	87.5 ± 2.12	0.707	85.7 ± 13.1	82.3 ± 12.8	0.614	100 ± 0.00	83.1 ± 12.4	0.208
Distribution										
Normal (≤140/90)	13 (81.3)	11 (78.6)	2 (100)	0.468	7 (77.8)	6 (85.7)	0.687	0 (0.00)	13 (86.7)	**0.032**
Abnormal (>140/90)	3 (18.8)	3 (21.4)	0 (0.00)		2 (2.22)	1 (14.3)		1 (100)	2 (13.3)	
Pulse pressure, BPM										
Mean ± SD	73.7 ± 8.50	72.8 ± 8.66	80.0 ± 4.24	0.276	75.3 ± 9.07	71.6 ± 7.85	0.398	65.0 ± 0.00	74.3 ± 8.46	0.307
Distribution										
Normal (60–100)	16 (100)	14 (100)	2 (100)	Undefined	9 (100)	7 (100)	Undefined	1 (100)	15 (100)	Undefined
Abnormal (<60 or >100)	0 (0.00)	0 (0.00)	0 (0.00)		0 (0.00)	0 (0.00)		0 (0.00)	0 (0.00)	
Donation duration, minutes										
Mean ± SD	5.48 ± 1.61	5.58 ± 1.70	4.78 ± 4.60	0.530	5.43 ± 1.50	5.53 ± 1.87	0.904	6.00 ± 0.00	5.44 ± 1.66	0.749
Distribution										
Normal (5–10)	10 (62.5)	9 (64.3)	1 (50.0)	0.696	5 (55.6)	5 (71.4)	0.515	1 (100)	9 (60.0)	0.424
Abnormal (<5 or >10)	6 (37.5)	5 (35.7)	1 (50.0)		4 (44.4)	2 (28.6)		0 (0.00)	6 (40.0)	

*Note*: Past infection (IgG+/IgM–) and recent infection (IgG–/IgM+ or IgG+/IgM+). The bold number was statistically significant in the *χ* ^2^ test or independent‐sample *T* tests (*p* < 0.05).

Abbreviations: BPM, beats per minute; DBP, diastolic blood pressure; SBP, systolic blood pressure.

There are important limitations to be considered when interpreting the results of this study. First, the small sample size of individuals exposed to SARS‐CoV‐2 limits our power to analyze and extrapolate the results to the whole population of Luanda, the capital city of Angola. Second, the effect of blood pressure changes was not taken into account for the different SARS‐CoV‐2 strains, which also deserves investigation in the future. Third, information about blood pressure changes during or after at least 6 months since SARS‐CoV‐2 exposure was not considered in this study and this should also be considered in future studies. Fourth, more sociodemographic (e.g., area of residence, occupation, educational level, and monthly income), behavioral (e.g., alcoholism, tobacco, and physical activities), and clinical (e.g., family history of chronic illness) information, were not investigated in this study. Therefore, future studies of this nature should also consider the possibility of including these variables that have considerable weight from the point of view of epidemiology and dissemination of infectious agents. Finally, the clinical outcome of individuals exposed to SARS‐CoV‐2 and who had high blood pressure was not recorded, therefore, our study presents very informative data and has many policy implications in the area of public health with the recent outbreak of the global pandemic. As we can see, as the COVID‐19 pandemic takes hold, with countless variants emerging, numerous research questions remain open, to which we draw the attention of the scientific community for the assessment of the impact of SARS‐CoV‐2 infection on the course or long term in populations from different settings, mainly in LMICs.^2^, ^16^ With the results of this study, we may in the future develop models to predict the cardiovascular outcome of patients with COVID‐19 based on data acquired during hospital admission. Also, our findings can contribute to the prediction of the risk of developing cardiovascular diseases based on evidence that can help in making appropriate decisions to ensure better clinical and hospital management of patients with COVID‐19. These results must be considered very preliminary, despite showing a possible influence of biological factors such as age, gender, and AB/Rh blood group in blood pressure indices among individuals with recent or past infection by SARS‐CoV‐2 in Angola, which could help to give a new direction to the management of individuals exposed to SARS‐CoV‐2 in Angola.

## 5 CONCLUSION

Our results showed significant variations in blood pressure indices of the Angolan population exposed to SARS‐CoV‐2. Individuals over 40 years and exposed to SARS‐CoV‐2 presented high blood pressure. Moreover, non‐O blood group individuals presented higher positivity to SARS‐CoV‐2 and high mean values of systolic, diastolic, and pulse blood pressure. This is a very preliminary study that suggests that careful follow‐up could be necessary for patients who have experienced SARS‐CoV‐2 infection and highlights the importance of follow‐up and monitoring for late events. However, assessing the impact on cardiovascular functions with ongoing or long‐term exposure to SARS‐CoV‐2 in individuals from resource‐limited countries, such as Angola, should be considered for further investigation in the future.

## AUTHOR CONTRIBUTIONS

**Cruz S. Sebastião**: Conceptualization; data curation; formal analysis; investigation; project administration; supervision; validation; writing—original draft; writing—review & editing. **Euclides Sacomboio**: Investigation; writing—review & editing. **Ngiambudulu M. Francisco**: Investigation; writing—review & editing. **Edson K. Cassinela**: Investigation; writing—review & editing. **António Mateus**: Project administration. **Zinga David**: Project administration. **Victor Pimentel**: Writing—review & editing. **Joana Paixão**: Project administration; writing—review & editing. **Jocelyne Neto de Vasconcelos**: Project administration; writing—review & editing. **Joana Morais**: Project administration; writing—review & editing.

## CONFLICT OF INTEREST STATEMENT

The authors declare no conflict of interest.

## ETHICS STATEMENT

The present study has been approved by the National Ethics Committee of the Angolan MoH (nr. 10/2021), the direction of the committee of the Instituto Nacional de Sangue (nr.726/GDG/INS/2020), and the executive board of the Clínica Girassol (nr.0841/GEPP/PCE/2021).

## TRANSPARENCY STATEMENT

The lead author Cruz S. Sebastião affirms that this manuscript is an honest, accurate, and transparent account of the study being reported; that no important aspects of the study have been omitted; and that any discrepancies from the study as planned (and, if relevant, registered) have been explained.

## Data Availability

All relevant data are within the article.
